# Mitochondria-Dependent Apoptosis of Con A-Activated T Lymphocytes Induced by Asiatic Acid for Preventing Murine Fulminant Hepatitis

**DOI:** 10.1371/journal.pone.0046018

**Published:** 2012-09-24

**Authors:** Wenjie Guo, Wen Liu, Shaocheng Hong, Hailiang Liu, Cheng Qian, Yan Shen, Xuefeng Wu, Yang Sun, Qiang Xu

**Affiliations:** State Key Laboratory of Pharmaceutical Biotechnology, School of Life Sciences, Nanjing University, Nanjing, China; Mayo Clinic, United States of America

## Abstract

Selectively facilitating apoptosis of activated T cells is essential for the clearance of pathogenic injurious cells and subsequent efficient resolution of inflammation. However, few chemicals have been reported to trigger apoptosis of activated T cells for the treatment of hepatitis without affecting quiescent T cells. In the present study, we found that asiatic acid, a natural triterpenoid, selectively triggered apoptosis of concanavalin A (Con A)-activated T cells in a mitochondria-dependent manner indicated by the disruption of the mitochondrial transmembrane potential, release of cytochrome *c* from mitochondria to cytosol, caspases activation, and cleavage of PARP. In addition, asiatic acid also induced the cleavage of caspase 8 and Bid and augmented Fas expression in Con A-activated T cells. However, following activation of T cells from MRL^lpr/lpr^ mice with mutation of Fas demonstrated a similar susceptibility to asiatic acid-induced apoptosis compared with normal T cells, suggesting that Fas-mediated death-receptor apoptotic pathway does not mainly contribute to asiatic acid-induced cell death. Furthermore, asiatic acid significantly alleviated Con A-induced T cell-dependent fulminant hepatitis in mice, as assessed by reduced serum transaminases, pro-inflammatory cytokines, and pathologic parameters. Consistent with the in vitro results, asiatic acid also induced apoptosis of activated CD4^+^ T cells in vivo. Taken together, our results demonstrated that the ability of asiatic acid to induce apoptosis of activated T cells and its potential use in the treatment of T-cell-mediated inflammatory diseases.

## Introduction

Immune responses are frequently characterized by major expansions of antigen-specific T cells that have potent effector functions. Apoptosis is an essential mechanism used to eliminate activated T cells during the shutdown process of excess immune responses and maintain proper immune homeostasis, while deficient apoptosis of activated T cells contributes to a wide variety of immune disorders and chronic inflammation [1]–[4]. Thus eliminate the unwanted activated T cells through facilitating apoptosis may provide a strategy for the treatment of T cell-dependent diseases. Such a strategy focusing on the pathogenic T cells may avoid intervention of normal immune responses to other foreign without affecting naive or non-activated T cells.

Three different death signaling pathways lead to apoptosis such as the extrinsic death receptor pathway, the intrinsic mitochondrion-dependent pathway and the intrinsic endoplasmic reticulum (ER) stress pathway [5]. These pathways work together to regulate the function of T cells [6]. Fas-mediated apoptosis can be regulated by the levels of Fas expression [7]. Stimulation of death receptors such as Fas or TNF-related apoptosis-inducing ligand receptors results in receptor aggregation and recruitment of the adaptor molecule Fas-associated death domain and caspase-8 [8]. Upon recruitment, caspase-8 becomes activated and initiates apoptosis by direct cleavage of downstream effector caspase such as caspase-3 [9]–[11]. The mitochondrial pathway is initiated by stress signals through the release of apoptogenic factors such as cytochrome *c*, or apoptosis inducing factor from the mitochondrial intermembrane space [12]. The release of cytochrome *c* into the cytosol triggers caspase-3 activation through formation of the cytochrome *c*/Apaf-1/caspase-9-containing apoptosome complex. Alternatively, small amounts of active caspase-8 cut Bid into a truncated form which induces the release of pro-apoptotic molecules from mitochondria [13]. Unfolded protein response and changes in calcium concentration may trigger ER stress which then induce caspase-12 activation, expression of GRP78 and CCAAT/enhancer-binding protein homologous protein (CHOP), and finally cause apoptosis [14].

**Figure 1:**
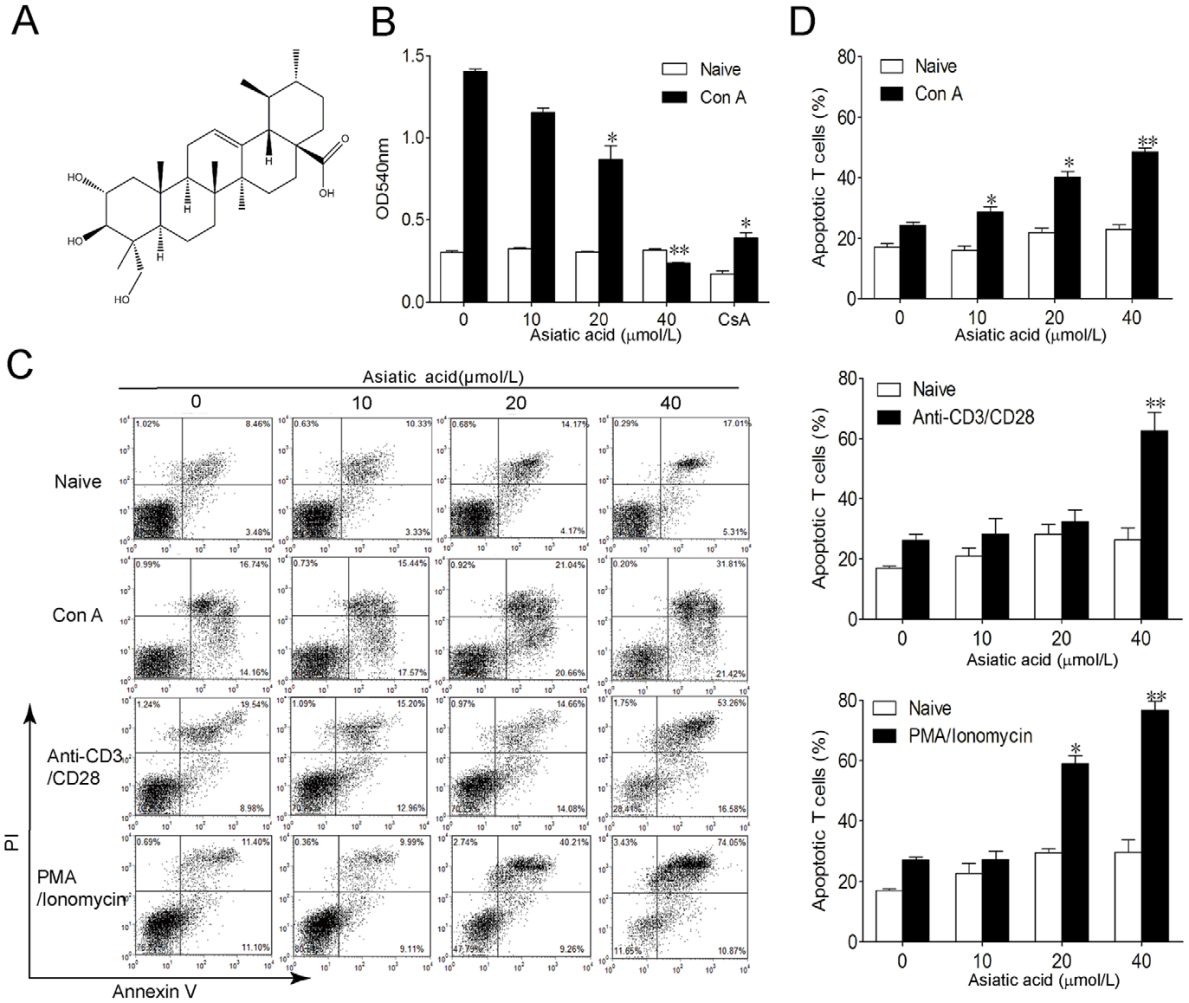
Asiatic acid induces apoptosis of activated T cells but not naïve T cells. (A) The chemical structure of asiatic acid. (B–D) Lymph node-derived T cells isolated from BALB/c mice were incubated with medium or in the presence of Con A (5 µg/ml) or anti-CD3/anti-CD28 (1 µg/ml) or PMA (100 ng/ml)/ionomycin (1 µg/ml) for 24 h. Then cells were further incubated with or without various concentrations of asiatic acid for 24 h. Cell proliferation was determined by MTT assay (B). Cells apoptosis were determined by Annexin V/PI staining (C). Data are shown as means ± SEM of three independent experiments (D). *P<0.05, **P<0.01 vs. drug-untreated group.

Asiatic acid, a natural triterpenoid compound, exhibits a variety of pharmacological activities including antioxidant, anti-inflammation, neuroprotective and anti-cancer effects [15]–[19]. However, the effect of asiatic acid on T cells remains unclear. In the present study, we demonstrate that asiatic acid alleviated Con A-induced T-cell-dependent fulminant hepatitis in mice by triggering apoptosis of activated T cells. Our data provide the molecular theoretical basis for clinical application of asiatic acid in patients with acute fulminant liver injury.

## Materials and Methods

### Ethics Statement

Animal welfare and experimental procedures were carried out strictly in accordance with the Guide for the Care and Use of Laboratory Animals (The Ministry of Science and Technology of China, 2006). All the animal experiments were approved by Nanjing University Animal Care and Use Committee and made to minimize suffering and to reduce the number of animals used.

**Figure 2:**
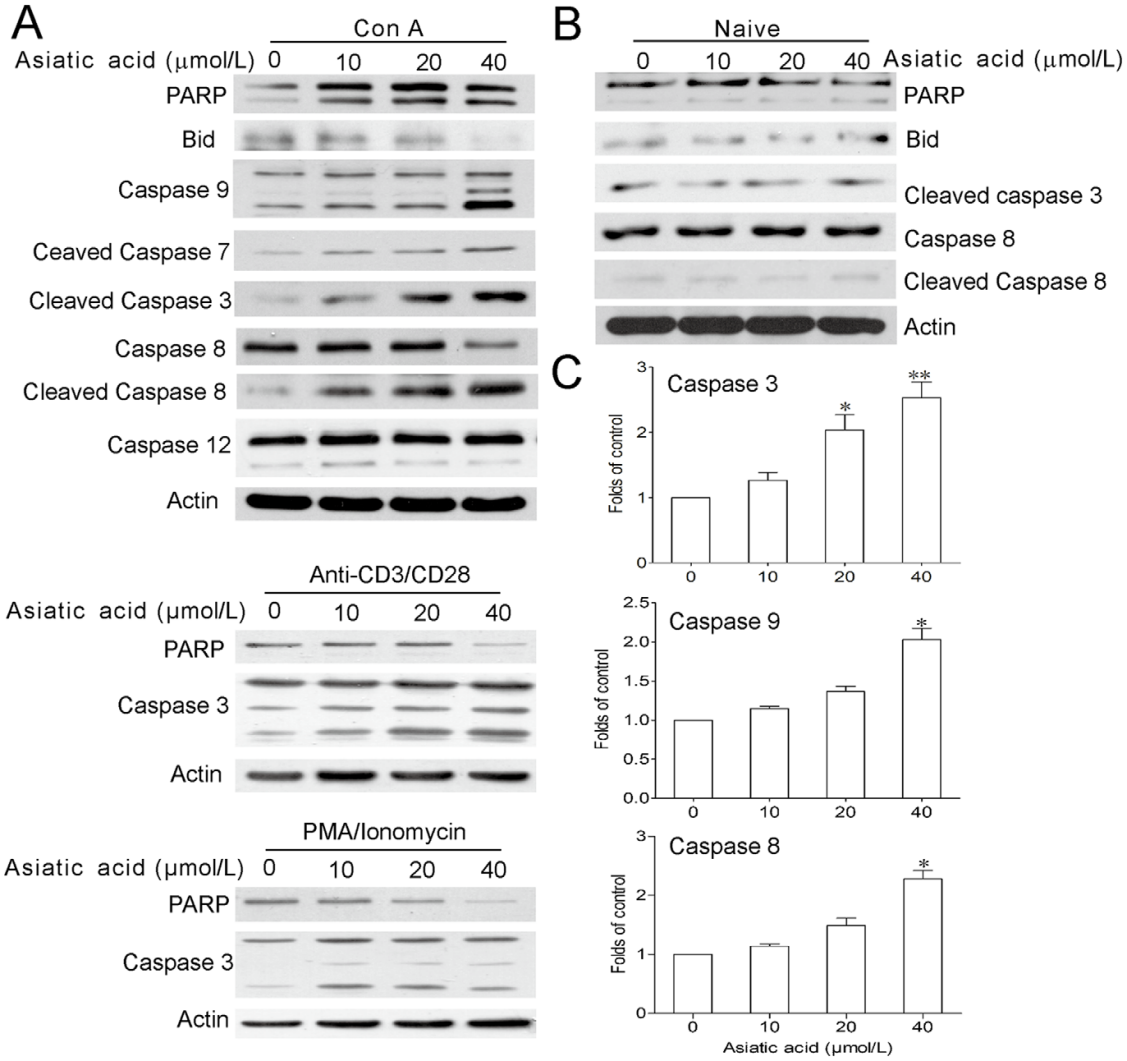
Asiatic acid results in caspases activation in activated T cells. (A–B) Lymph node-derived T cells isolated from BALB/c mice were incubated in medium or in the presence of Con A (5 µg/ml) or anti-CD3/anti-CD28 (1 µg/ml) or PMA (100 ng/ml)/ionomycin (1 µg/ml) for 24 h. Then cells were further incubated with or without various concentrations of asiatic acid for 24 h. Protein levels of Bid, caspase-3, -9, -7, -8 and -12, and PARP were examined by Western blotting. (C) Enzymatic activities of caspase-3, -9, and -8 of Con A-activated T cells after asiatic acid treated for 12 h were determined. Data are shown as means ± SEM of three independent experiments. *P<0.05 vs. drug-untreated group.

### Animals

Specific pathogen-free, 8–10-week-old female BALB/c mice were purchased from Experimental Animal Center of Jiangsu Province (Nanjing, China). Eight-week-old female MRL^lpr/lpr^ mice with a C57BL/6 background were purchased from Model Animal Research Center of Nanjing University (Nanjing, China).

### Drugs, Reagents and Antibodies

Asisatic acid, Concanavalin A (Con A), Cyclosporin A (CsA), thapsigargin (TG) and dexamethasone was purchased from Sigma Aldrich (St. Louis, MO). JC-1 was purchased from Invitrogen (Carlsbad, CA). Kits for determining serum alanine transaminase (ALT), aspartate transaminase (AST) were purchased from Nanjing Jiancheng Bioengineering Institute (Nanjing, China). Annexin V/PI Kit was purchased from Jingmei Biotech (Nanjing, China). ELISA kits for tumor necrosis factor-α (TNF-α) and interferon-γ (IFN-γ) were purchased from Dakewe Biotechnology Company (Shenzhen, China). Anti-CD3, anti-CD28 and anti-Bid were purchased from BD Pharmingen (San Diego, CA). Caspase-3, -8 and -9 activity assay kits were purchased from Beyotime Institute of Biotechnology (Nantong, China). PE-anti-mouse Fas mAb was purchased from eBioscience (San Diego, CA). Anti-full length and cleaved caspase-3/-8/-9/-7/-12, anti-CHOP and anti-poly (ADP-ribose) polymerase (PARP) were purchased from Cell Signaling Technology (Beverly, MA). Anti-cytochrome *c*, anti-COX IV, anti-GRP78, anti-Actin and anti-α Tubulin were purchased from Santa Cruz Biotechnology (Santa Cruz, CA). Mouse T cell enrichment column was purchased from R&D system (Minneapolis, MN). The cells were incubated in RPMI 1640 medium supplemented with 100 U/mL of penicillin, 100 µg/mL of streptomycin and 10% fetal calf serum under a humidified 5% (v/v) CO_2_ atmosphere at 37°C. All other chemicals were obtained from Sigma Aldrich (St. Louis, MO).

**Figure 3:**
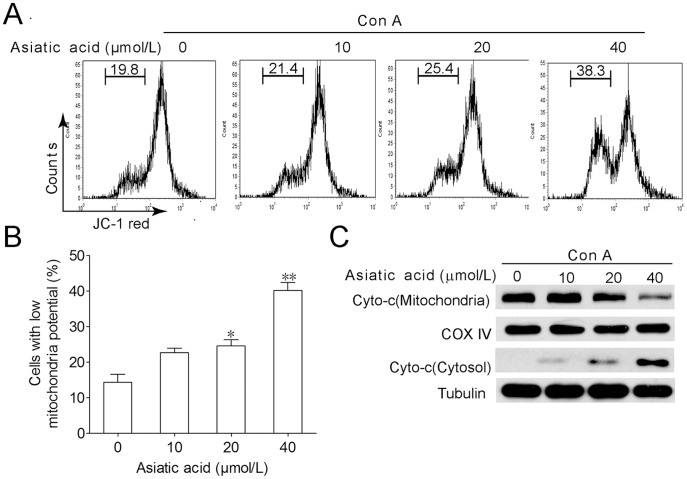
Asiatic acid triggers apoptosis of Con A-activated T cells through mitochondrial pathway. Lymph node-derived T cells isolated from BALB/c mice were incubated in medium or in the presence of Con A (5 µg/ml) for 24 h. Then cells were further incubated with or without various concentrations of asiatic acid for 24 h. Cells were stained with JC-1 intensity of FL-2 was analyzed by ﬂow cytometry to determine mitochondrial membrane potential. The cells located with low red fluorescence were considered as low mitochondrial transmembrane potential (A). Data are shown as means ± SEM of three independent experiments (B). *P<0.05, **P<0.01 vs. drug-untreated group. (C) The release level of cytochrome *c* from mitochondria was examined by Western blotting. The results shown are representative of three experiments.

### Cell Viability Assay

Lymph node-derived T cells isolated from BALB/c mice were activated by Con A (5 µg/ml) or anti-CD3/anti-CD28 (1 µg/ml) or PMA (100 ng/ml)/ionomycin (1 µg/ml) for 24 h, which were indicated as activated cells, whereas those without stimulation were used as non-activated (naive) cells. The cells were further incubated in 96 well-plate with or without various concentrations of asiatic acid at a density of 3×10^5^ cells/well for 24 h. For MTT assay, 20 µl of MTT (Sigma; 4 mg/ml in PBS) was added per well 4 h before the end of the incubation. MTT formazan production was dissolved by DMSO replacing the medium. The optical density at 540 nm (OD_540_) was measured by a microplate reader.

**Figure 4:**
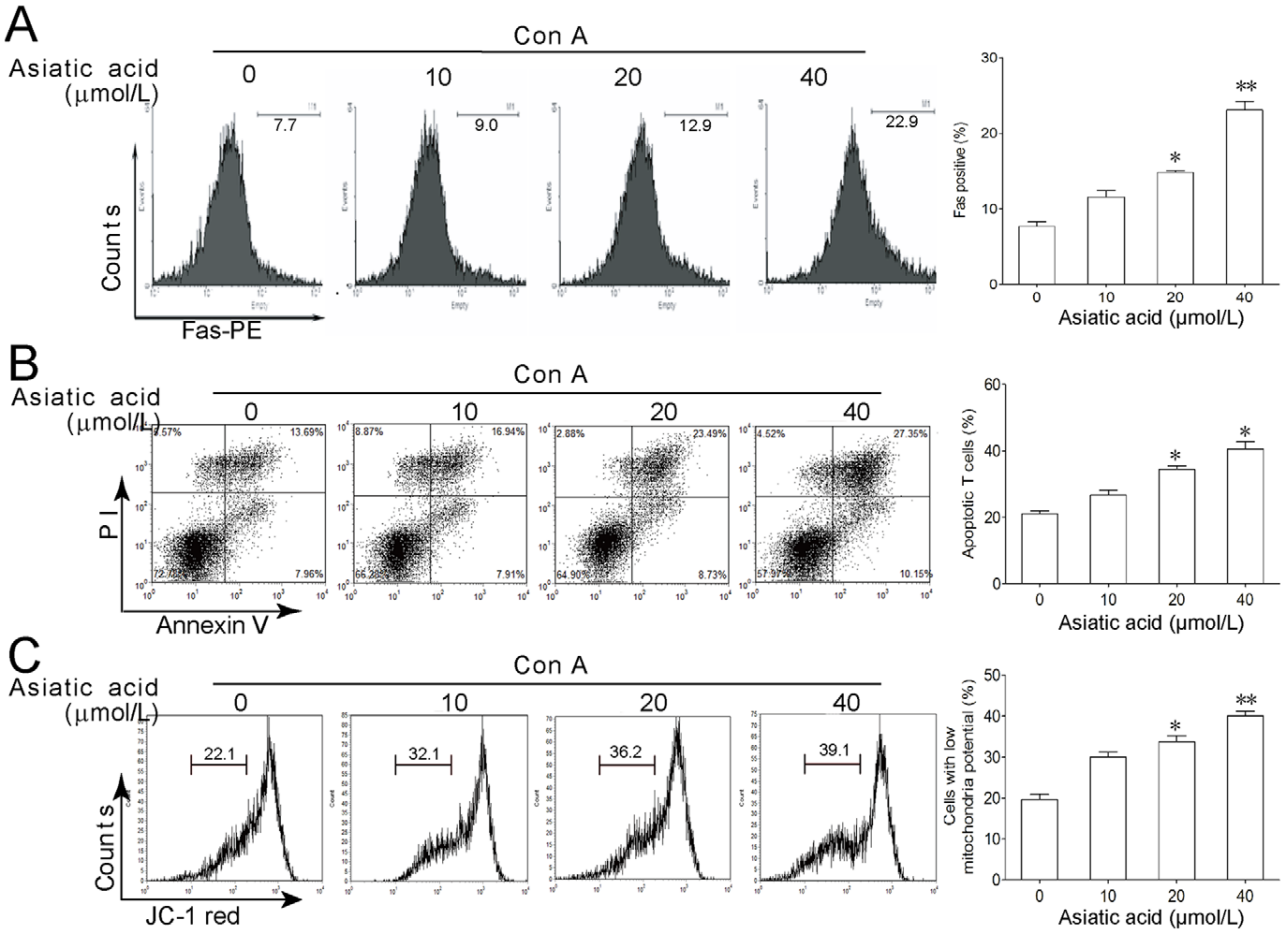
Fas-mediated death-receptor apoptotic pathway does not mainly contribute to asiatic acid-induced cell death of activated T cells. (A) Lymph node-derived T cells isolated from BALB/c mice were incubated in medium or in the presence of Con A (5 µg/ml) for 24 h. Then cells were further incubated with or without various concentrations of asiatic acid for 24 h. Fas expression at the surface of T cells was evaluated by flow cytometry. (B, C) Lymph node-derived T cells isolated from MRL^lpr/Lpr^ mice were incubated in medium or in the presence of Con A (5 µg/ml) for 24 h. Then cells were further incubated with or without various concentrations of asiatic acid for 24 h. Apoptosis and mitochondria membrane potential were evaluated by Annexin V-FITC/PI (B) and JC-1 staining (C), respectively. Data are shown as means ± SEM of three independent experiments. *P<0.05, **P<0.01 vs. drug-untreated group.

### Cell Apoptosis Assay

Cell apoptosis was determined by Annexin V/PI staining. The cells were measured by flow cytometry after addition of FITC-conjugated Annexin V and PI as previously described [20]. Annexin V^+^/PI^-^ and Annexin V^+^/PI^+^ cells were considered as apoptotic cells in the early and late phase, respectively.

### Western Blot

Western blot was done as described before [21]. Briefly, the cells were collected and lysed in lysis buffer containing protease inhibitor (protease inhibitor cocktail, Pierce). The proteins were fractionated by SDS-PAGE and electrotransferred to PVDF membranes. Different antibodies were used for blotting, and detection was done by LumiGLO chemiluminescent system (KPL, Guildford, UK).

**Figure 5:**
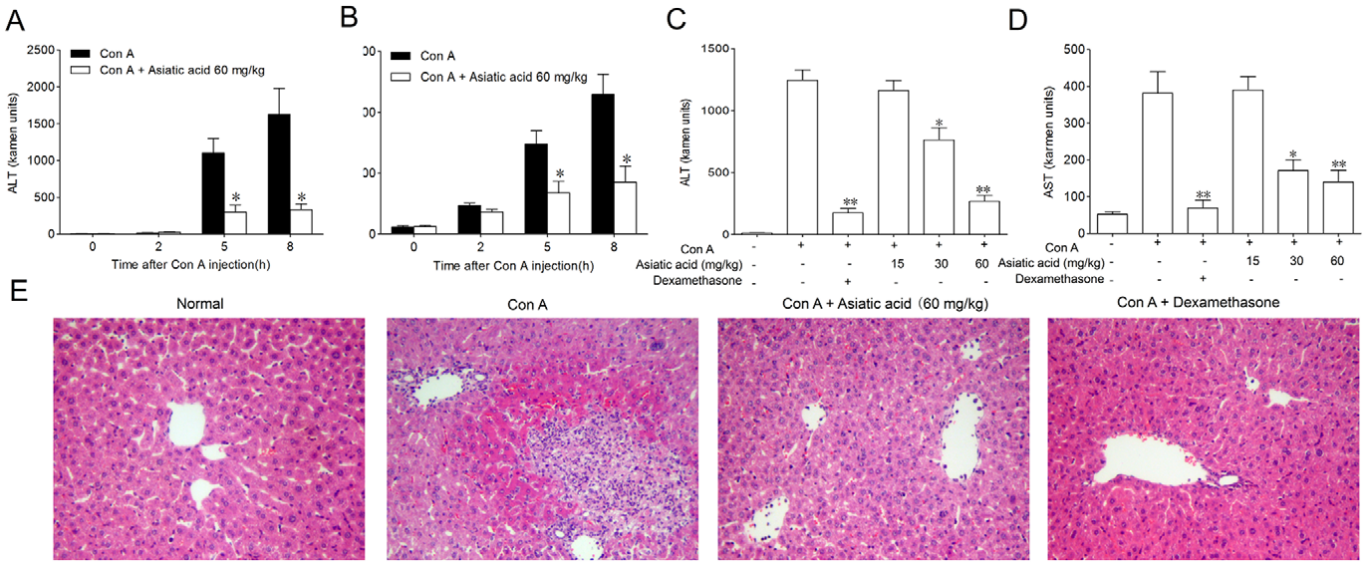
Asiatic acid protects against Con A-induced T-cell-mediated acute fulminant hepatitis in mice. BALB/c mice were received an intravenous Con A injection (15 mg/kg body weight). In the drug treatment group, asiatic acid (intragastric administration) and dexamethason (intramscular injection) were given twice at 8 h before and 1 h after Con A injection, respectively. (A, B) Time course of serum alanine transaminase (ALT) and aspartate transaminase (AST) release. Values are shown as the means ± SEM of three mice at each time point. *P<0.05 vs. Con A group at the same time point. (C, D) Dose-dependent inhibition on ALT and AST release. Values are shown as the means ± SEM of eight mice in each group. *P<0.05, **P<0.01 vs. Con A group. (E) Photomicrographs of representative mouse livers with H&E staining are shown (original magnification ×100).

### Mitochondrial Transmembrane Potential Assay

Mouse lymph node-derived T cells were cultured in the presence or absence of 5 mg/ml of Con A for 24 h and then incubated with several doses of asiatic acid for 6 h. The disruption of mitochondrial transmembrane potential was measured using JC-1 staining by flow cytometry as previously reported [20].

### Caspase Activity Assay

Cells were lysed in the lysis buffer containing 10 mmol/L HEPES, 5 mmol/L dithiothreitol, 2 mmol/L EDTA, 1 mM phenylmethylsulfonyl fluoride, and 0.1% CHAPS, pH 7.4. After 12,000 *g* centrifugation for 15 min, the protein concentration in the supernatant was determined by a BCA protein assay kit. Caspase activity was determined following the instruction of commercial kit (Beyotime, Nantong, China).

**Figure 6:**
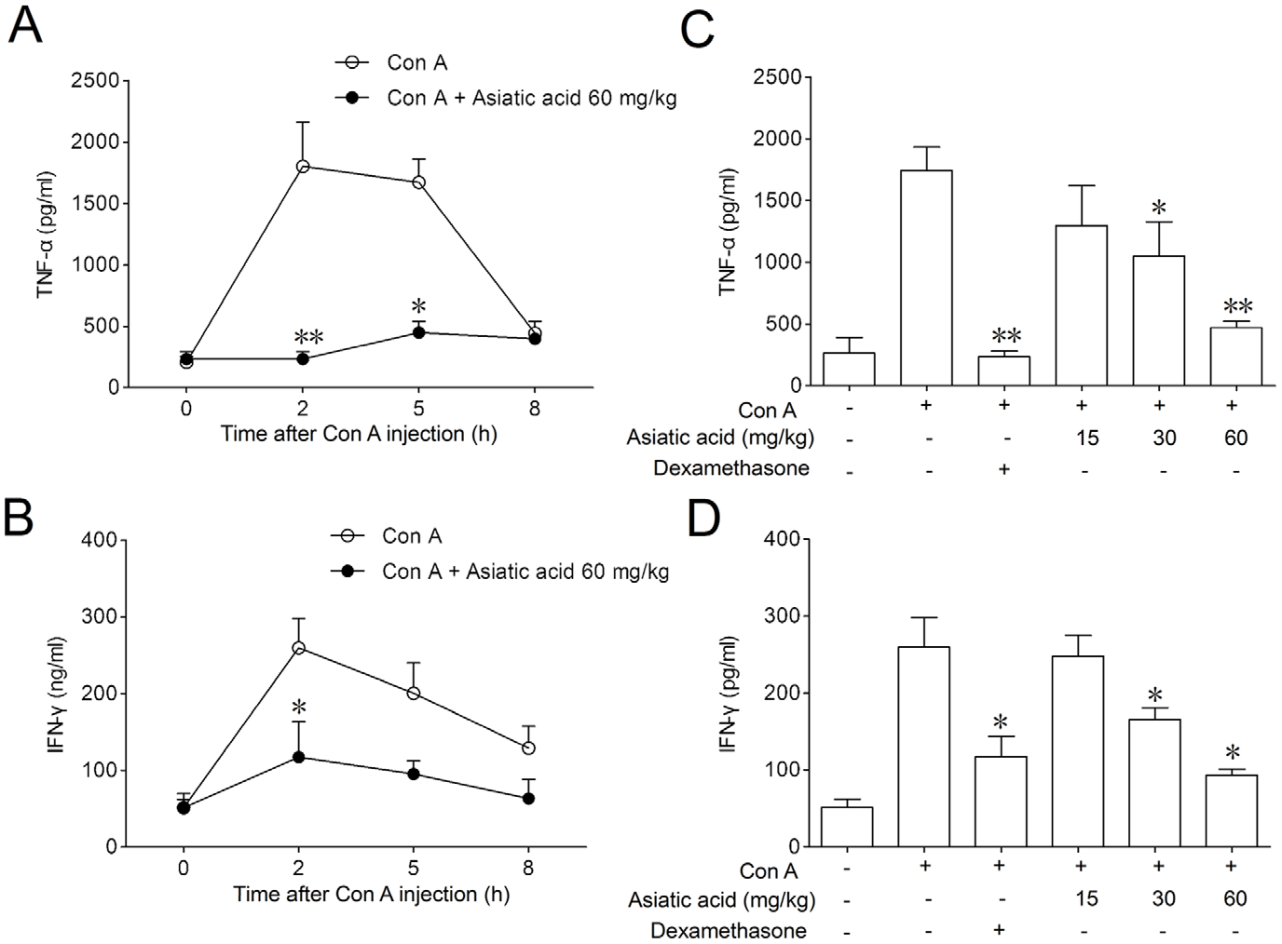
Asiatic acid inhibited inhibits TNF-α and IFN-γ levels in serum from Con A-induced hepatitis in mice. Cytokine levels in serum were measured by ELISA. (A, B) Time course of TNF-α and IFN-γ released into serum. Values are shown as the means ± SEM of three mice at each time point. *P<0.05, **P<0.01 vs. Con A group at the same time point. (C, D) Dose-dependent inhibition on TNF-α and IFN-γ levels. Values are shown as the means ± SEM of eight mice in each group. *P<0.05, **P<0.01 vs. Con A group.

### Con A-induced T-cell-dependent Hepatitis

Specific pathogen-free, 8–10-week-old female BALB/c mice were purchased from Experimental Animal Center of Jiangsu Province (Jiangsu, China). They were maintained with free access to pellet food and water in plastic cages at 21±2°C and kept on a 12 h light/dark cycle. Acute liver injury was induced by injecting mice with Con A in pyrogen-free phosphate buffered saline (PBS) at 20 mg/kg via the tail vein. In the drug treatment group, asiatic acid (intragastric administration) and dexamethasone (intramscular injection) were given twice at 8 h before and 1 h after Con A injection, respectively. In control animals, the same dose of PBS was given before Con A injection. Mice were killed at the indicated time points, and blood samples and livers were collected. Serum alanine transaminase (ALT) and aspartatetransaminase (AST) activities as well as cytokine levels were measured by commercial kits as the protocols indicated. For the dose-dependent experiments, there were eight mice in each group, while for the time point experiments, three mice were sacrificed.

**Figure 7:**
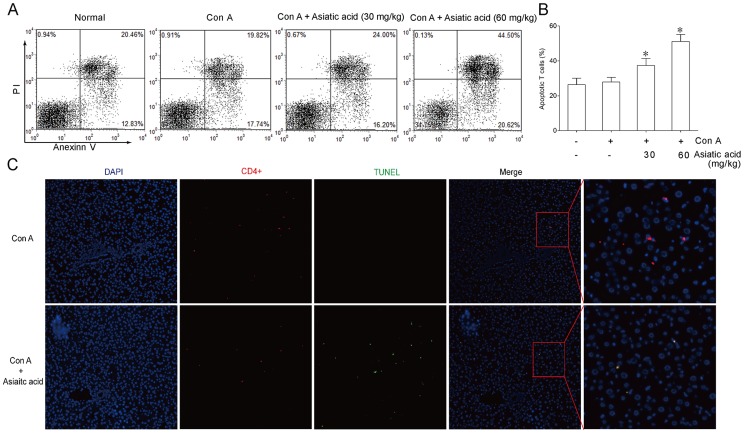
Asiatic acid induces apoptosis of activated T cells in Con A-induced T-cell-mediated hepatitis. BALB/c mice were received an intravenous Con A injection (15 mg/kg body weight). Asiatic acid (30 and 60 mg/kg) were intragastric given twice at 8 h before and 1 h after Con A injection, respectively. Splenic T cells were isolated at 2 h after Con A administration. After culture for 24 h, cells were co-stained with Annexin V/PI to determine apoptosis by flow cytometry (A). Values are shown as the means ± SEM of eight mice (B). *P<0.05, **P<0.01 vs. Con A group. (C) Paraffin-embedded liver tissue sections from vehicle- and asiatic acid-treated group were stained for TUNEL-FITC and CD4-PE. The nuclei were stained with DAPI. Original magnification, ×100.

### TUNEL Assay

For the detection of apoptosis, paraffin-fixed liver tissue sections were stained by the terminal deoxynucleotidyl transferase (TdT)-mediated dUTP nick end labeling (TUNEL) technique using an in situ apoptosis detection kit (Vazyme Biotech Co., Ltd., Nanjing, China) according to the manufacturer’s instructions. Sections were co-stained with CD4^+^-PE and DAPI and observed under a fluorescence microscope (BX51, Olympus, Tokyo, Japan).

### Cytokine Assay

Blood samples were obtained from mice at the indicated time points and immediately centrifuged at 1500 *g* for 15 min. Serum samples were stored at −70°C until ready for used. Serum levels of IFN-γ and TNF-α were determined using QuickEIA™ ELISA kits from Dakewe Biotechnology Company (Shenzhen, China).

### Flow Cytometric Analysis

Cells were stained with antibodies diluted in PBS containing 2% fetal calf serum and 0.1% NaN_3_ then analyzed by FACS Calibur ﬂow cytometer (Becton Dickinson, San Jose, CA).

**Figure 8:**
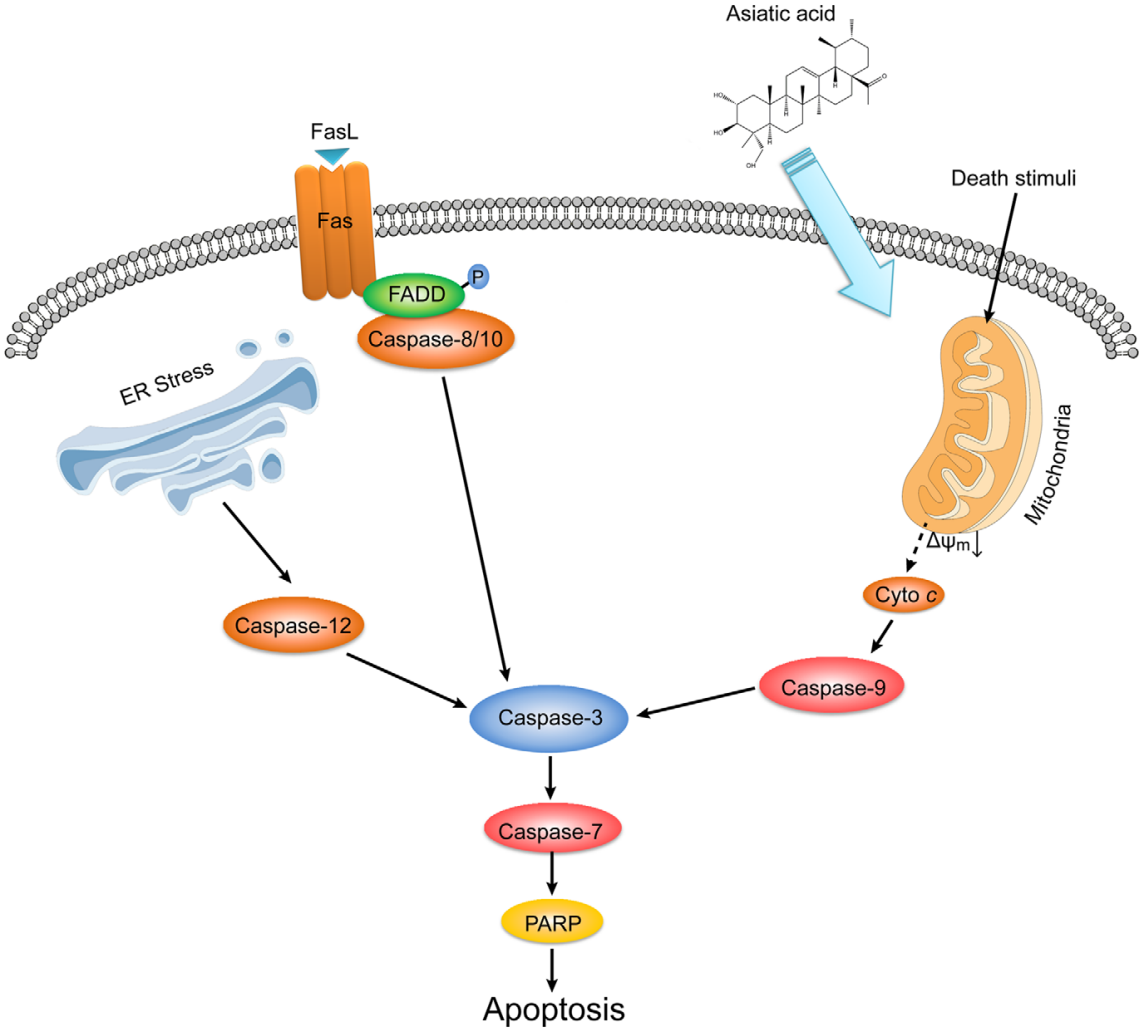
Overview of cell death pathways for asiatic acid-induced apoptosis in Con A-activated T cells. Asiatic acid triggered apoptosis of Con A-activated T cells in a mitochondria-dependent manner indicated by the disruption of the mitochondrial transmembrane potential, release of cytochrome *c* from mitochondria to cytosol, caspase-9 and caspase-3 activation and cleavage of PARP. At the same time, following activation of T cells from MRL^lpr/lpr^ mice with mutation of Fas demonstrated a similar susceptibility to asiatic acid-induced apoptosis compared with normal T cells. In addition, asiatic acid hardly influenced caspase-12 activation and the expression of GRP78 and CHOP. Taken together, these results suggest that Fas-mediated death-receptor apoptotic pathway and endoplasmic reticulum (ER) stress-mediated apoptotic pathway do not mainly contribute to asiatic acid-induced cell death.

### Statistical Analysis

All results shown represent means ± SEM from triplicate experiments performed in a parallel manner unless otherwise indicated. Statistical analyses were performed using a one-way analysis of variance (ANOVA), followed by two-tailed Student’s *t* test. The significance of difference is indicated as *P*<0.05 or *P*<0.01.

## Results

### Asiatic Acid Inhibited Proliferation and Induced Apoptosis of Activated T Cells

In order to examine the effect of asiatic acid on T cells, lymph node-derived T cells isolated from BALB/c mice were incubated in medium or in the presence of Con A or anti-CD3/anti-CD28 or PMA/ionomycin for 24 h. Then cells were further incubated with or without various concentrations of asiatic acid for 24 h. As shown in Fig. 1B, asiatic acid dose-dependently inhibited the proliferation of Con A-activated T cells without influencing quiescent T cells. However, the immunosuppressant cyclosporine A (CsA) inhibited cell viability both in Con A-activated T cells and quiescent T cells (Fig. 1B). Furthermore, Annexin V/PI staining showed that asiatic acid markedly induced apoptosis of T cells activated by various methods in a dose-dependent manner (Fig. 1C and 1D). For example, the apoptotic rate of Con A-activated T cells increased from 25.5±1.1% (drug-untreated group) to 50.3±1.3% (40 µmol/L asiatic acid treated group). Notably, there was minor but not significant increase of cell apoptosis in naive T cells, which the apoptotic rate increased from 17.0±1.2% (drug-untreated group) to 26.9±7.2% (40 µmol/L asiatic acid treated group). These results suggest that asiatic acid mainly induces apoptosis of activated T cells rather than naive T cells.

### Asiatic Acid Induced Activation of Caspases and Cleavage of PARP in Activated T Cells

To further investigate the molecular mechanism of asiatic acid-induced apoptosis, we monitored the caspases which take part in the apoptosis process as in Fig. 2. After asiatic acid treatment in Con A-activated T cells, the full length of caspase-3, -9, -7, and -8 but not caspase-12 were cleaved to their active forms, accompanied with the elevation of their enzyme activity (Fig. 2C). PARP and caspase-3 cleavage were also observed in anti-CD3/anti-CD28 or PMA/ionomycin-activated T cells after asiatic acid treatment (Fig. 2A). On the contrary, there was almost no change on the activation or expressions of PARP, caspase-8, -3 or Bid in naive T cells treated by asiatic acid (Fig. 2B).

### Asiatic Acid Induced Apoptosis of Con A-activated T Cells through Mitochondrial Pathway

As shown in Fig. 3A and 3B, asiatic acid treatment disrupted the mitochondrial transmembrane potential of Con A-activated T cells demonstrated by decreased JC-1 red fluorescence. With the collapse of mitochondrial transmembrane potential, the release of cytochrome *c* form mitochondrion to cytosol was greatly increased in a dose-dependent manner (Fig. 3C). These results indicate that mitochondrial apoptotic pathway is involved in asiatic acid-induced apoptosis in activated T cells.

### Death-receptor Apoptotic Pathway did not Mainly Contribute to Asiatic Acid-induced Cell Death in Con A-activated T Cells

As shown in Fig. 2A, asiatic acid treatment induced the cleavage of caspase 8 and Bid in Con A-activated T cells. In addition, asiatic acid also augmented Fas expression in activated T cells (Fig. 4A). These results indicate that death-receptor pathway may be also involved in asiatic acid-induced apoptosis. However, following activation the T cells from MRL^lpr/lpr^ mice with mutation of Fas demonstrated a similar susceptibility to asiatic acid-induced apoptosis and collapse of mitochondria membrane potential compared with normal T cells (Fig. 4), suggesting that Fas-mediated death-receptor apoptotic pathway does not mainly contribute to asiatic acid-induced cell death.

Furthermore, we wonder whether endoplasmic reticulum (ER) stress-mediated apoptotic pathway is involved in asiatic acid-induced cell death. Expressions of the key makers of ER stress, GRP78, CHOP and caspase-12 were assessed by Western blot. As shown in Fig. S1, asiatic acid did not influence the expressions of GRP78, CHOP or activation of caspase-12, while our positive control thapsigargin significantly enhanced their expressions. These results suggest that ER stress-mediated apoptotic pathway is not involved in asiatic acid-induced cell death.

### Asiatic Acid Protected Mice from Con A-induced T-cell-mediated Acute Fulminant Hepatitis by Inducing Apoptosis of Activated T Cells

For determining whether asiatic acid affected T cells in vivo in the same manner it does in vitro, Con A-induced T-cell-mediated acute hepatitis model was used in the present study. Intravenous administration of Con A resulted in a time-dependent increase in the serum ALT and AST levels. A significant elevation was detected 5 h after Con A administration and the peak was reached at 8 h. Results from Fig. 5A and 5B indicated that 60 mg/kg of asiatic acid significantly reduced the ALT and AST levels at 5 h and 8 h, respectively. Eight hours after Con A injection, asiatic acid reduced the serum levels of ALT and AST in a dose-dependent manner (Fig. 5C and 5D). The histological examination of liver sections showed a massive necrosis with cytoplasmic swelling, inflammatory infiltration and severe hepatocyte degeneration in the Con A-treated mice without medication (Fig. 5E). Against these changes, pretreatment with 60 mg/kg of asiatic acid markedly reduced the extent of liver damage. TNF-α and IFN-γ play a casual role in the onset of liver damage [22], [23]. Compared with the significant increase in serum TNF-α and IFN-γ levels due to Con A injection, asiatic acid exhibited obvious inhibition in a time- and dose-dependent fashion (Fig. 6). In freshly isolated splenic T cells from asiatic acid-treated mice, the apoptotic counts were significantly higher than those from untreated mice during the in vitro incubation period (Fig. 7A and 7B). Moreover, TUNEL-FITC and CD4-PE co-staining analysis showed that TUNEL positive cells were mostly colocalized with liver CD4^+^ T cells in asiatic acid-treated mice (Fig. 7C), suggesting that asiatic acid induces apoptosis of intrahepatic CD4^+^ T cells in Con A-induced hepatitis in mice.

## Discussion

Most of the activated T cells need to be deleted after the antigenic stimulus is removed. Specific induction of pathogenic T cell apoptosis is beneficial in depressing excess immune responses and maintaining immune homeostasis, and represents a new approach for future treatment of numerous T-cell-mediated immune diseases. However, few drug candidates have been reported through such selective induction of apoptosis of activated pathogenic T cells thus far. Herein we reported that a natural triterpenoid, asiatic acid, mainly induced apoptosis of activated T cells rather than naive T cells in vitro. Furthermore, the facilitation of apoptosis by asiatic acid in activated T cells was remarkably effective in blocking the development of T-cell-dependent fulminant hepatitis, indicating that this small molecule might be a potential lead compound useful in treating T-cell-mediated liver disorders in human beings.

Upon examination of apoptotic pathways, we observed the upregulation of Fas, truncation of Bid, activations of caspase-8, -9, -7, -3 and the disruption of mitochondria membrane potential induced by asiatic acid, indicating that death-receptor and mitochondrial apoptotic pathways are both involved in asiatic acid-induced cell death. However, using T cells from MRL^lpr/lpr^ mice with mutation of Fas, a similar susceptibility to asiatic acid-induced apoptosis was seen compared with normal T cells, suggesting that Fas-mediated death-receptor apoptotic pathway does not mainly contribute to asiatic acid-induced cell death. In addition, it was not likely that ER stress-mediated apoptotic pathway was involved in asiatic acid-induced cell death, because asiatic acid treatment failed to induce the expression of ER stress-related proteins such as GRP78 and CHOP as well as activation of caspase-12 in activated T cells. Taken together, our data strongly suggest asiatic acid triggers mitochondria-dependent apoptotic cell death in activated T cells.

Increasing evidence suggests that T-cell-mediated immunity is one of the dominant causes in a variety of liver diseases involving autoimmune and viral hepatitis and T-cell activation is a critical initial step in the pathogenesis of liver damage [24]. In mice, T-cell-dependent hepatitis can be modeled using in vivo administration of Con A, a plant lectin and T-cell mitogen. This mitogen induces polyclonal T-cell activation in vitro and causes severe immune-mediated hepatitis characterized by increased serum levels of transaminases and infiltration of peripheral T cells into the liver [24]–[26]. These findings indicate that activated T cells play a detrimental role in liver injury. Therefore, effective elimination of pathogenic effector T cells has been a therapeutic strategy for the treatment of T-cell-mediated liver diseases [20]. However, there is still lacking chemical candidates which selectively targeting pathogenic activated T cells in hepatitis. In this study, asiatic acid significantly alleviated Con A-induced hepatitis with an almost recovery from the elevation of serum transaminase levels at the dose of 60 mg/kg. Splenic T cells isolated from mice in asiatic acid-treated group showed a higher potential to apoptosis than those from vehicle-treated group, indicating a higher sensitivity of activated peripheral T cells to apoptosis by asiatic acid in vivo. It coincided with our in vitro result that activated T cells by Con A showed a higher percentage of apoptosis after asiatic acid treatment. Moreover, TUNEL-FITC and CD4-PE co-staining analysis showed that TUNEL positive cells were mostly colocalized with liver CD4^+^ T cells in asiatic acid-treated mice, suggesting that asiatic acid induces apoptosis of intrahepatic CD4^+^ T cells in Con A-induced hepatitis in mice. Consequently, these effects of asiatic acid may result in less activated peripheral T cell infiltration into the liver and then less liver damage. Previously, asiatic acid was reported to protect hepatocytes from various injuries [27], [28], suggesting the hepatocyte protective effect of asiatic acid. In the present study, we demonstrated that asiatic acid could induce apoptosis of Con A-activated T cells both in vivo and in vitro, which provides one of the possible mechanisms that how asiatic acid protects Con A-induced murine fulminant hepatitis. Frankly, we think that both the induction of T cell apoptosis and the protective effect on hepatocytes by asiatic acid will contribute to the amelioration of liver injury induced by Con A.

Previous studies have shown that some herb extracts or small compounds can selectively induce activated T cells to apoptosis. For instance, an extract of Dregea volubilis as well as soyasapogenol A and saikosaponin a are reported to inhibit the proliferation and activation of T cells through induction of apoptosis [29]–[31]. KE-298, an anti-rheumatic drug, specially augments apoptosis of activated T cells in rheumatoid arthritis by decreasing the expression of X-linked inhibitor-of-apoptosis protein [32]. A recent report demonstrates that fraxinellone-induced T cell apoptosis in murine fulminant hepatitis correlates with down-regulation of anti-apoptotic cellular FLICE-inhibitory protein expression [20]. However, chemicals that selectively augment apoptosis of activated T cells for the treatment of T-cell-mediated diseases have not been well validated. In the present study, we showed that asiatic acid, a natural triterpenoid, exerted a unique immunosuppressive action in hepatitis by increasing the release of cytochrome *c* form mitochondrion to cytosol, then subsequently enhancing intrinsic apoptotic pathways in Con A-activated T cells, showing a distinct mechanism from the aforementioned small compounds. Most importantly, in the present study, the activated T cells induced by Con A in vitro accounted for most of the pathogenic T cells which induced T cell-mediated hepatitis in vivo. However, other small compounds mentioned above triggered apoptosis of activated T cell stimulated by anti-CD3, or phorbol/inomycin, which were not the actual etiopathogenisis of their in vivo animal model of immune diseases, including experimental allergic encephalitis, adjuvant arthritis, and rheumatoid arthritis. Specially, it is noted that asiatic acid, having little effect on non-activated T cells, promoted apoptosis in activated T cells, which avoided the disadvantage of non-specific immunosuppression. This raises the possibility that asiatic acid may be especially useful for eliminating activated pathogenic T cells that contribute to T cell-related immune diseases.

In conclusion, the purpose of this study is to report a novel strategy for hepatitis therapy involving the triggering of apoptosis of activated T cells by means of a natural triterpenoid. Our results also indicate that it might be feasible to treat T-cell-mediated liver injury using reagents like asiatic acid by facilitating apoptosis of detrimental effector T cells without affecting naive T cells. Based on the results of the present study, the mechanism by which asiatic acid induces apoptotic cell death in activated T cells is summarized in Fig. 8.

## Supporting Information

Figure S1 **Effects of asiatic acid on relevant molecules in ER stress-mediated pathway.** Lymph node-derived T cells isolated from BALB/c mice were incubated in medium or in the presence of Con A (5 µg/ml) for 24 h. Then cells were further incubated with or without various concentrations of asiatic acid and thapsigargin (TG) for 24 h. Protein level of GRP78 and CHOP were examined by Western blotting. The results shown are representative of three experiments.
(TIF)
