# Supplementary figures and images for: Mitochondria-Dependent Apoptosis of Con A-Activated T Lymphocytes Induced by Asiatic Acid for Preventing Murine Fulminant Hepatitis
Source: PLoS One. 2012 Sep 24;7(9):e46018. doi: 10.1371/journal.pone.0046018 (PMC3454338; doi:10.1371/journal.pone.0046018)

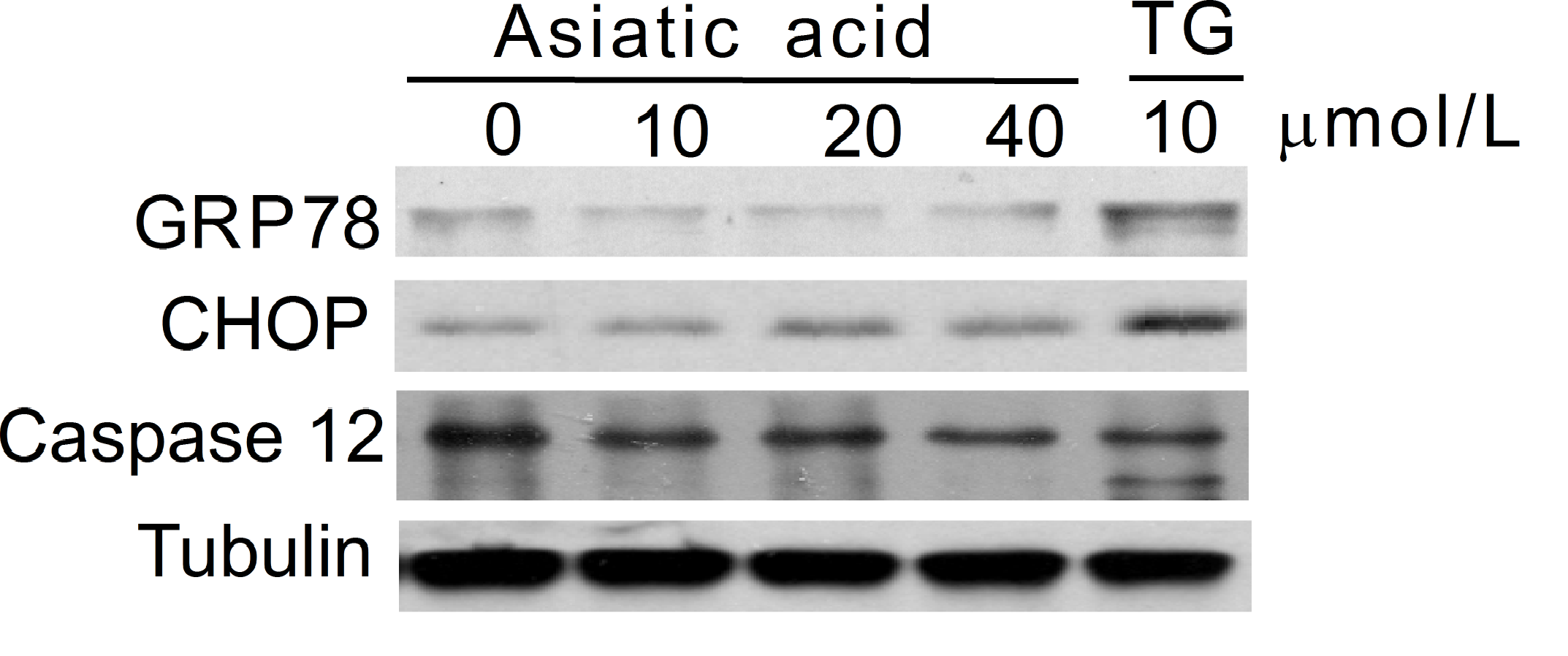

Supplement: Figure S1 — Effects of asiatic acid on relevant molecules in ER stress-mediated pathway. Lymph node-derived T cells isolated from BALB/c mice were incubated in medium or in the presence of Con A (5 µg/ml) for 24 h. Then cells were further incubated with or without various concentrations of asiatic acid and thapsigargin (TG) for 24 h. Protein level of GRP78 and CHOP were examined by Western blotting. The results shown are representative of three experiments. (TIF) [file pone.0046018.s001.tif]
